# A high-resolution polarimeter formed from inexpensive optical parts

**DOI:** 10.1038/s41598-020-61715-7

**Published:** 2020-03-25

**Authors:** A. J. Harvie, T. W. Phillips, J. C. deMello

**Affiliations:** 1 Department of Chemistry, NTNU, Trondheim, Norway; 2 Department of Chemistry, Imperial College London, London, United Kingdom

**Keywords:** Chemistry, Optics and photonics

## Abstract

We describe a high resolution laser polarimeter built from commodity optical components. The optical rotation angle is determined by measuring the phase difference between two harmonically modulated polarised laser beams – an ‘object beam’ that passes through the sample under test and a ‘reference beam’ that bypasses the sample. The complete polarimeter may be assembled from low cost off-the-shelf parts for less than £300 (UK Sterling). Data acquisition and analysis are carried out on a microcontroller running an efficient algorithm based on the sliding Discrete Fourier Transform. Despite its low cost, the polarimeter is a fully automatic, research-grade instrument with an accuracy of ±0.0013° and a precision of ±0.0028° – comparable to far costlier commercial instruments. The polarimeter’s ease of use, compact size, fast measurement times and high angular resolution make it a capable and versatile tool for analytical science, while its low cost means it is ideally suited for use in resource-constrained environments and process monitoring. The polarimeter is released here as open hardware, with technical diagrams, a full parts list, and source code for its firmware included as Supplementary Information.

## Introduction

The tremendous advances in electronic hardware over recent decades have led to substantial improvements in the speed, accuracy, precision and functionality of scientific instrumentation. The electromechanical automation of intricate physical and chemical procedures, the development of brighter and more stable light-sources, the proliferation of low-noise sensors, improvements in amplifier technology, and the availability of better signal processing and data analysis techniques (to name but a few) have all contributed positively to analytical performance. Taking the subject of this manuscript – polarimetry – as an example, virtually all polarimeters in the early 1960s were manually operated instruments with hand-rotated analysers and telescopic eye-pieces that relied on human judgement to determine the angle of rotation. Today, they are motorised instruments with integrated photodetectors that automatically measure optical rotation angles at the push of a button. However, while functionality has improved tremendously over the past fifty years, equipment costs have changed very little: in 1965, a manually operated high-resolution polarimeter from a leading manufacturer cost US$3300, equivalent to $27000 now; today, automatic high resolution polarimeters from the same supplier range from $14000 to $38000.

It is broadly the case that instrumentation manufacturers have favoured improvements in functionality over reductions in cost. Consequently, much routine laboratory equipment remains beyond the financial reach of many potential users, especially those in developing countries. At the same, time there is an increasing need to integrate high performance instrumentation into chemical reactors for the purposes of in-line screening, optimisation and quality assurance, which is typically feasible only if the instruments are substantially less expensive than the reactors themselves. Hence, there is a growing demand for instruments that offer near state-of-the-art performance at a much lower price level.

The high cost of current scientific instrumentation is partly due to an absence of the intense downward price pressures that exist for commodity hardware and consumer devices. But it is also due in many cases to the use of costly precision-engineered mechanical or optical parts that are not amenable to low-cost manufacturing. Typical components in a modern polarimeter, for instance, include prismatic polarisers, Faraday rotators, goniometers, and high-resolution rotary encoders, which add substantially to the parts cost. However, by using alternative measurement procedures that circumvent the need for high-end components (or at least permit the use of cheaper, lower-specified components), instrumentation costs may be reduced considerably. Timing-based measurements are particularly well suited to low cost implementation due to the high accuracy of standard crystal oscillators^1^. In addition, ratiometric and differential measurements – in which a reference signal is measured alongside the target signal – can significantly lessen hardware demands by compensating for fluctuations or drift in experimental parameters^2^. By combining such techniques with low cost microcontrollers or system-on-chip computer systems (which integrate hardware for data acquisition and digital signal processing into a single package), considerable simplifications may often be achieved in the experimental set-up without sacrificing analytical performance. Hence, through the careful selection of measurement and data analysis procedures, significant opportunities exist for substantial cost savings in many analytical measurements.

This paper is specifically concerned with developing an affordable, high performance chemical polarimeter for studying the behaviour of chiral crystals, molecules and molecular assemblies. Key applications include molecular identification and quantitation, determination of enantiomeric excess, monitoring of reaction kinetics, and enantioselective column chromatography^3^. In common with other polarimeters, fields of use include the pharmaceutical industry, the food industry, the cosmetics industry, the chemical industry, forensic science, and medicine.

High-end commercial polarimeters typically use frequency-based null-point measurements to determine the rotation angle of a sample under test^4^. In a typical implementation, the polarisation plane of a probe beam is aligned perpendicular to the axis of a polarising element (the “analyser”), and the transmitted light intensity is recorded using a photodetector. A Faraday oscillator of frequency *f* perturbs the polarisation plane of the probe beam a few degrees either side of its ‘resting’ position, causing the signal recorded by the photodetector to be modulated at a frequency 2 *f*. Insertion of an optically active sample between the Faraday oscillator and the polariser generates an additional signal at a frequency *f*, proportional in size to the angle of rotation. By means of a servo loop the analyser is rotated until the *f*-signal is eliminated and a new null-position is reached. Hence, by measuring the angular rotation required to restore the null-condition, the optical rotation is determined.

Implemented in this way, the null-point method delivers typical resolutions of ±0.002° for rotation angles smaller than one degree, deteriorating to ±0.2% for larger rotations (see Supplementary Table S2). There have also been a few reports of developmental polarimeters with angular resolutions at the ten-microdegree level^5–7^, but these have variously required stringent alignment procedures, thorough vibration isolation, complex and costly experimental set-ups, and/or temperature stabilisation of various components, suggesting they are still some way from commercialisation, and in any case may not be appropriate for routine analytical testing. In practice millidegree accuracy is the commercial state of the art, and an appropriate target for a low-cost instrument.

While frequency-based null-point measurements offer high accuracy and fast measurement times of a few seconds, their reliance on precision-engineered optomechanical parts make them a costly solution for polarimetry, with limited scope for cost reductions. In this paper, we exploit an alternative approach to polarimetry that uses a differential-phase-based detection procedure to measure the rotation angle. Using only low-cost off-the-shelf components, the entire system may be constructed for < £300 (UK Sterling). Despite its low cost the system offers comparable performance to high-end commercial instruments, with a fixed accuracy of ±0.0013° and a precision of ±0.0028° at all rotation angles. Owing to its combination of low cost and research-grade performance, the instrument is likely to be of particular interest to those working in heavily resource-constrained environments. In addition – as an inexpensive, compact and fully automated system – it should be well suited to process monitoring and reaction optimisation.

## Experimental Setup

The method used here is based on a principle originally proposed by Tumerman^8^, in which the rotation angle is determined by measuring the phase difference between two harmonically modulated polarised laser beams – an ‘object beam’ that passes through the sample under test and a ‘reference beam’ that bypasses the sample. Importantly, the method requires no precision-engineered optomechanical parts, relying instead on a timing-based procedure to determine the optical rotation angle. The specific optical set-up we use follows an optimised configuration proposed by Vishnyakov *et al*.^9^, with a number of component substitutions to reduce costs and a simple addition of an extra photodetector to compensate for laser drift. The principal changes are: the replacement of a 632.8 nm HeNe laser by a 650 nm laser diode; the replacement of a prism beam-splitter by a plate beam-splitter; the replacement of Glan-Thomson prism polarisers by thin-film polarisers; and the replacement of a personal computer (PC) equipped with a 24-bit data acquisition card by a low cost microcontroller running an optimised signal-processing algorithm. These simple changes allow us to bring the bill-of-materials below £300, without compromising instrumental performance.

**Figure 1:**
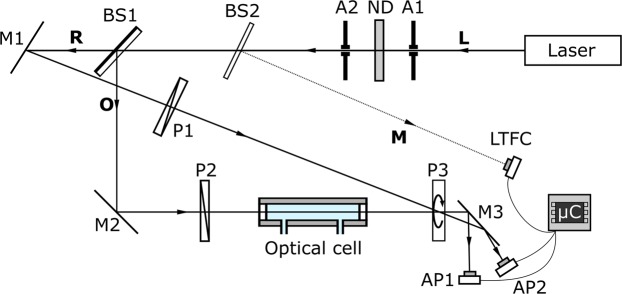
Schematic of optical setup. The beam (**L**) from a laser diode is passed through a 1-mm aperture (A1), an optional neutral density filter (ND), and a second 1-mm aperture (A2). A 50:50 plate-type beam-splitter (BS1) divides the laser beam into an object beam (**O**) and a reference beam (**R**). The reference beam is directed by a plane mirror (M1) through a fixed thin-film polariser (P1) onto a rotatable thin-film polariser (P3), which is mounted on a hollow-shaft motor. The object beam is directed by a plane mirror (M2) through a fixed thin-film polariser (P2); it then passes through a 5-cm optical cell, before striking the same point on the rotating polariser. The object and reference beams pass through the centre of P3 and are then directed by a third mirror (M3) onto a pair of amplified photodiodes (AP1 and AP2). P3 is rotated at a fixed angular frequency *f*, causing the intensity of light striking the photodiodes to be modulated at a frequency 2 *f*. The laser intensity is continuously monitored by means of a second beam-splitter (BS2), which directs a weak monitor beam (**M**) onto a light-to-frequency converter (LTFC). The digital signal from the LTFC and the analogue signals from the two amplified photodiodes are measured using a microcontroller (µC). The microcontroller analyses the input signals and generates a digital output signal equal to the phase-difference between the object and reference channels.

The optical set-up is shown schematically in Fig. 1. A laser beam from a 650 nm, 1 mW laser diode (Acculase PWM-650-1-S, Global Laser) is passed sequentially through a 1-mm “beam-cleaning” aperture (A1), a neutral-density filter (ND, [optical density = 1, NE510B-A, Thorlabs]), and a second 1-mm aperture (A2). The cleaned laser beam strikes a 50:50 plate-type beam-splitter (BS1, [Thorlabs]) which divides it into separate object (O) and reference (R) beams of similar intensity. The reference beam is directed by a plane mirror (M1) through a fixed thin-film polariser (P1, [22 CA 25, Comar Optics]); it then passes through the centre of an identical thin-film polariser (P3), which acts as a rotatable analyser. The analyser is centrally mounted on a hollow-shaft brushless motor (Turnigy 4008, 56 KV) that allows the laser beam to pass through unimpeded. The object beam is directed by a plane mirror (M2) through a fixed thin-film polariser (P2) and a 5-cm optical cell (see Supplementary Fig. S1 for details), before it too passes through the centre of the rotating analyser. The motor is driven at a fixed speed of approximately 480 rpm by an electronic speed controller (DECS 50/5, Maxon), causing both beams to be modulated at twice the frequency of rotation (see below). The modulated beams are reflected by a third plane mirror (M3) onto two amplified photodiodes (AP1 and AP2, [OPT101, Texas Instruments]), and the photodiode output signals are measured and processed using a microcontroller (μC, [Teensy 3.6, PJRC]) with two independent analogue-to-digital converters (ADCs). Introducing an optically active sample into the object path changes the phase difference between the two channels by an amount equal to twice the optical angle of rotation. Hence, two measurements of phase difference are sufficient to determine the angle of rotation – an “active” measurement with an optically active sample in the object path and a “blank” measurement without the sample present.

In a small modification to the optical set-up used by Vishnyakov *et al*. a glass slide (BS2) located just after the second beam-cleaning aperture is used to divert a small percentage of the laser light to a photodetector, allowing the intensity of the laser beam to be monitored in real-time. The object and reference signals are divided by the measured laser intensity prior to data analysis to correct for drift in the laser intensity. Since the microcontroller’s two ADCs are permanently dedicated to measuring the object and reference signals, the laser intensity is monitored using a light-to-frequency converter (LTFC) connected to a digital pin of the microcontroller. Data analysis is carried out within the microcontroller, meaning the entire circuitry for measuring and processing the signals comprises just two amplified photodiodes, one light-to-frequency converter and a microcontroller development board. Photographs of the assembled polarimeter are shown in Supplementary Figs. S2 and S3, while a schematic of the detection circuitry is shown in Supplementary Fig. S4. A full parts list is given in Supplementary Table S1.

### Principle of operation

The operating principle of the polarimeter may be understood by considering how the rotation of the analyser (P3) relative to the two fixed polarisers (P1 and P2) affects the transmission of the reference and object beams, taking into account our use of imperfect thin-film polarisers which do not fully polarise the incident light.

If unpolarised light is incident on an imperfect polariser, the polarisation *P* of the transmitted light is given by $P=({I}_{1}-{I}_{2})/({I}_{1}+{I}_{2})$, where *I*_1_ and *I*_2_ are the transmitted intensities of polarised light parallel and perpendicular to the transmission axis of the polariser. For two similar polarisers positioned in a line with their axes oriented at an angle *θ* to one another, the overall transmittance of a polarised light beam is given by the following equation (a modification of Malus’ law)^10^:

1 $$T(\theta )=\left(\frac{1}{1+P}\right)+\left(\frac{{P}^{2}}{1+P}\right)\cos (2\theta )=T(0)+\Delta T(\theta )$$

where $T(0)$ is the mean transmittance and $\Delta T(\theta )$ is an angle-dependent correction. If the analysing polariser is rotated at an approximately constant angular velocity *ω* about the optical axis, $\Delta T$ will vary co-sinusoidally with the time *t* at twice the angular velocity:

2 $$\Delta T(t)\propto \,\cos (2\theta )\propto \,\cos \,[2(\omega t+{\theta }_{0}+{\tilde{\theta }}_{t})]$$

where ${\theta }_{0}$ is the angle between the polariser axes at $t=0$ and ${\tilde{\theta }}_{t}$ is an unknown time-dependent term that corresponds to jitter in the rotation speed. Inserting an optically active component between the polarisers has the effect of introducing a further phase-shift $2\Delta \theta$ into the measured signal where $\Delta \theta$ is the angle of rotation. Hence, we may write for the reference beam, the blank object beam, and the active object beam the following three equations:

3a $$\Delta {T}_{{\rm{ref}}}(t)\propto \,\cos \,[2(\omega t+{\theta }_{{\rm{ref}}}^{0}+{\tilde{\theta }}_{t})]$$

3b $$\Delta {T}_{{\rm{blk}}}(t)\propto \,\cos \,[2(\omega t+{\theta }_{{\rm{obj}}}^{0}+{\tilde{\theta }}_{t})]$$

3c $$\Delta {T}_{{\rm{act}}}(t)\propto \,\cos \,[2(\omega t+{\theta }_{{\rm{obj}}}^{0}+\Delta \theta +{\tilde{\theta }}_{t})]$$

from which it follows directly that the associated phases are:

4a $${\phi }_{{\rm{ref}}}=2({\theta }_{{\rm{ref}}}^{0}+{\tilde{\theta }}_{t})$$

4b $${\phi }_{{\rm{blk}}}=2({\theta }_{{\rm{obj}}}^{0}+{\tilde{\theta }}_{t})$$

4c $${\phi }_{{\rm{act}}}=2({\theta }_{{\rm{obj}}}^{0}+\Delta \theta +{\tilde{\theta }}_{t})$$

Note, Eq. (1) is trigonometrically equivalent to Eq. (24) in ref. ^10^. For two perfect polarisers with *P* = 1, Eq. (1) reduces to Malus’ law$:\,T=\frac{1}{2}(1+\,\cos (2\theta ))={\cos }^{2}\theta$.

Hence, calculating the phase of the blank and active object beams relative to the reference beam, we obtain:

5a $$\Delta {\phi }_{{\rm{blk}}}={\phi }_{{\rm{blk}}}-{\phi }_{{\rm{ref}}}=\,2({\theta }_{{\rm{obj}}}^{0}-{\theta }_{{\rm{ref}}}^{0})$$

5b $$\Delta {\phi }_{{\rm{act}}}={\phi }_{{\rm{act}}}-{\phi }_{{\rm{ref}}}=2({\theta }_{{\rm{obj}}}^{0}+\Delta \theta -{\theta }_{{\rm{ref}}}^{0})$$

which, when subtracted, yield the required relationship:

6 $$\Delta \theta =\frac{1}{2}(\Delta {\phi }_{{\rm{act}}}-\Delta {\phi }_{{\rm{blk}}}).$$

Hence, the angle of rotation may be determined by first measuring the phase difference between the blank object beam and the reference beam, and then measuring the phase difference between the active object beam and the reference beam, taking care not to disturb the alignment of the polarisers between the two measurements.

### Some observations on the experimental procedure

We make several observations about the experimental procedure. Firstly, the signals from the two amplified photodiodes are divided by the signal from the light-to-frequency converter prior to data analysis, yielding scaled signals that are linearly proportional to the transmittances of the two beams. This differs from the original procedure used by Vishnyakov *et al*. (who used the unscaled signals for data processing), and eliminates laser drift as a potential source of measurement inaccuracy. Importantly, the modified procedure allows us to replace the HeNe laser used in the original set-up by an inexpensive laser diode with no optical feedback, substantially reducing the cost of the optical set-up. Secondly, our use of low cost thin-film polarisers in place of costly Glan-Thomson prism polarisers does not compromise measurement performance. This is evident from Eq. (1), which shows that a sinusoidal modulation of the transmittance will occur irrespective of the *P*-value of the polarisers (with *P* affecting only the amplitude and DC offset of the sinusoids). Thirdly, since both the reference and the object beams pass through a common spot on the same rotating analyser, variations in the direction of the plane of polarisation across the area of the analyser do not affect the measured signals. Fourthly, for the same reason, the phase jitter 2 ${\tilde{\theta }}_{t}$ due to non-uniform motor movement is the same for both channels and therefore cancels out when measuring $\Delta {\phi }_{{\rm{blk}}}$ and $\Delta {\phi }_{{\rm{act}}}$, eliminating a potential source of measurement error. Finally, since all required information is extracted directly from the measured harmonic signals there is no need to separately monitor the angular velocity of the motor, which beneficially avoids the need for a rotary encoder or other form of velocity meter. Taken together, these features of the analysis procedure mean it possible to achieve accurate measurements of the angle of optical rotation, even with low cost opto-mechanics.

### Calculating the phase difference

The phase difference between the object and reference channels is determined here using the discrete Fourier transform (DFT)^11^ – an approach that benefits from good noise rejection and low computational demands. Vishnyakov *et al*. reported an effective three-step procedure for calculating the phase difference between the object and reference channels, involving a forward DFT, a filtering step, and an inverse DFT, with the two DFTs being calculated via the Fast Fourier Transform (FFT)^12^. Here we use a simpler analysis procedure based on interpolation of the DFT phase spectra to the calculated frequency of the signals, which avoids the need to carry out the inverse DFT. We further amend our procedure to use a version of the DFT known as the sliding DFT (sDFT)^13,14^, which allows the necessary calculations to be carried out in real-time on a low-cost microcontroller. In contrast to the FFT, which calculates the full DFT in a single shot once data acquisition has been completed, the sDFT spreads the computational effort over the full data acquisition cycle, with the DFT being updated every time a new data point is acquired. The phase difference is calculated automatically by the software provided (see Supplementary Information). If you are not interested in the mathematical details of the calculation, you may proceed directly to the section entitled ‘Experimental Implementation’.

For ease of explanation, we begin by describing the analysis procedure as carried out using the FFT (Method 1). Consider a digitised function $y(0),\,y(1),\,y(2),\ldots y(N-1)$ obtained by sampling a continuous function $y(t)$ at the discrete times ${t}_{0},\,{t}_{1},\,{t}_{2}\ldots {t}_{N-1}$ where ${t}_{i}=i\Delta t$. The DFT decomposes the digitised signal into *N* complex oscillators of frequency ${f}_{k}=k{f}_{s}/N$, phase ${\phi }_{k}$ and real magnitude *R*_*k*_, where $k=0,1,2\ldots N-1$ and ${f}_{s}=1/\Delta t$ is the sample rate. Defining the complex magnitude of each oscillator as $Y(K)={R}_{k}{e}^{i{\phi }_{k}}$, the DFT may be written as a sequence of *N* complex numbers $Y(0),\,Y(1),Y(2),\ldots Y(N-1)$ where

7 $$Y(k)={R}_{k}{e}^{i{\phi }_{k}}=\mathop{\sum }\limits_{n=0}^{N-1}y(n){e}^{-i2\pi kn/N}$$

and ${R}_{k}={\rm{abs}}(Y(k))$ and ${\phi }_{k}=\text{arg}(Y(k))$^11^. The DFT is usually determined by means of the fast Fourier transform – a computationally efficient method for determining all *N* frequency bins in a single ‘shot’. However it should be noted that, in situations where only a subset of the frequency bins is required, the FFT is not necessarily the most efficient approach.

The DFT treats the input data $y(0)\ldots y(N-1)$ as if it were a single period of a periodic signal, which is true only if the acquisition time $N\Delta t$ is an exact multiple of the signal’s time period *T*^11^. If this condition is met, the harmonics of the input signal coincide exactly with the frequency bins of the DFT, meaning the harmonic frequencies may be read directly from the peaks in the DFT magnitude spectrum. In the more typical situation where $N\Delta t$ is not exactly divisible by *T*, the harmonic frequencies of the input signal fall in-between the bins of the DFT, and interpolation methods must be used to estimate the harmonic frequencies. Furthermore, due to the end-point discontinuity between $y(0)$ and $y(N-1)$, each harmonic peak is spread across the entire DFT spectrum – an effect known as ‘spectral leakage’^15^ – preventing reliable signal analysis.

Spectral leakage may be reduced by multiplying the input signal by a *hump-*shaped window function $w(n)$ prior to calculating the DFT^15^, which brings the initial and final values of the input signal to approximately zero and so reduces the magnitude of the end-point discontinuity. Here we use the Hann function $w(n)=0.5[1-\,\cos (2\pi n/(N+1))]$ which, as discussed below, is a convenient choice for the sDFT^16,17^. Following windowing, the resulting DFT magnitude spectrum exhibits a broadened peak around each harmonic (rather than a delta function when $N\Delta t$ is exactly divisible by *T*). For a mono-sinusoidal input, a single broadened peak is observed in the magnitude spectrum, and the (non-integer) signal frequency $\hat{k}$ may be estimated by interpolation using the following relationship^18^:

8 $$\hat{k}={k}^{\ast }+2\left[\frac{R({k}^{\ast }+1)-R({k}^{\ast }-1)}{2R({k}^{\ast })+R({k}^{\ast }-1)+R({k}^{\ast }+1)}\right]$$

where k^*^ corresponds to the largest peak in the magnitude spectrum. Once $\hat{k}$ has been determined, the phase of the signal may be straightforwardly obtained by interpolating the phase spectrum to $\hat{k}$.

**Figure 2:**
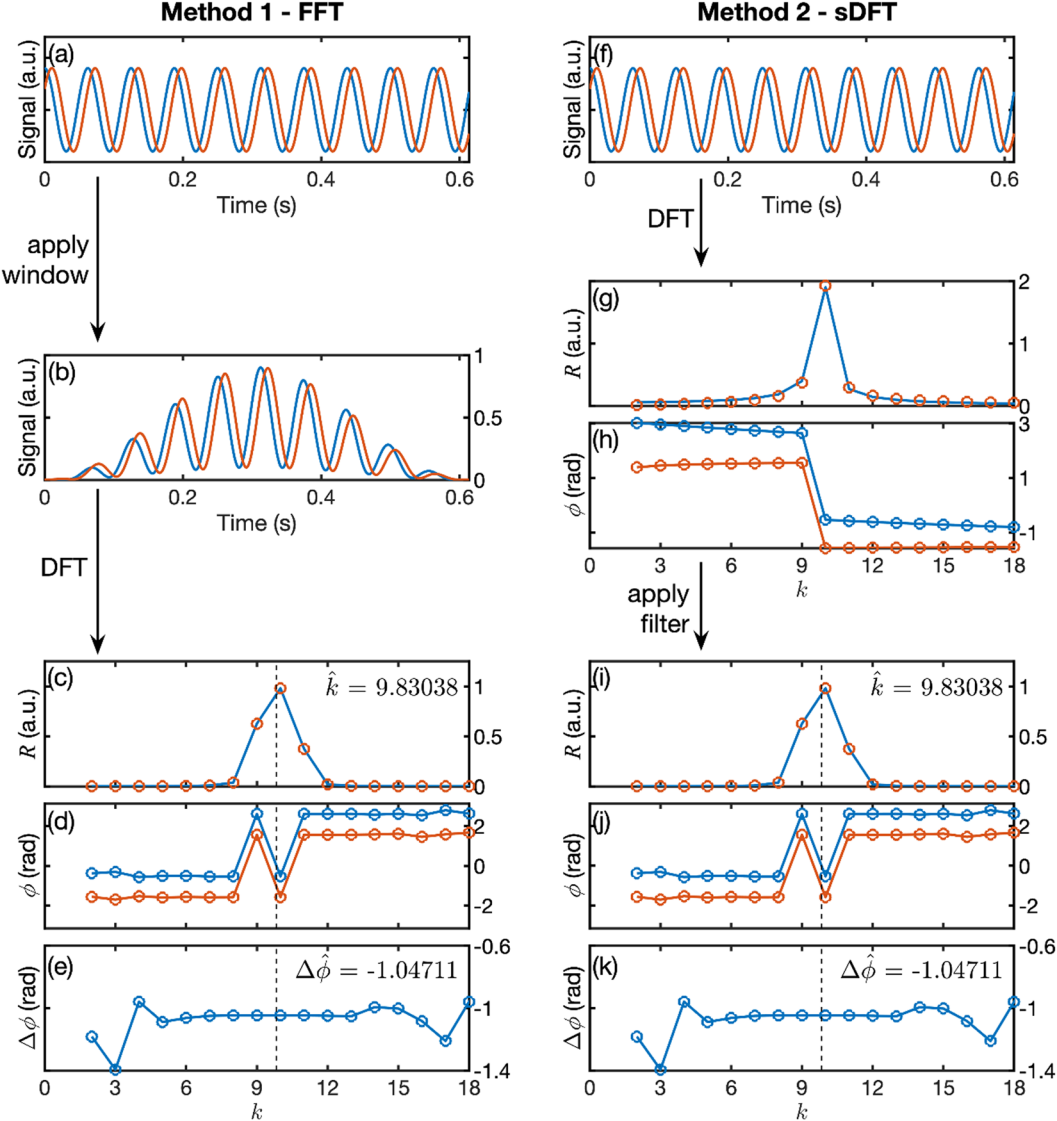
**(a–e)** Use of the FFT with time-domain windowing to determine the phase difference between two mono-sinusoidal signals. (**a**) Simulated 12-bit reference (red) and object (blue) signals, with a phase difference of $\,{\phi }_{{\rm{obj}}}-{\phi }_{{\rm{ref}}}=-\,\pi /3$. (**b**) Input signals after time-domain windowing using the Hann function. (**c**) DFT magnitude spectra of windowed input signals, calculated using the FFT. The vertical line at $k=9.83038$ denotes the mean signal frequency extracted from the DFT using Eq. (8). (**d**) DFT phase-spectra of windowed input signals, calculated using the FFT. (**e**) Differential phase-spectrum $\Delta \phi ={\phi }_{{\rm{obj}}}-{\phi }_{{\rm{ref}}}$, wrapped to lie in the range $-\pi  < \Delta \phi \le \pi$. The phase-difference $\Delta \phi =-\,1.04711$ is obtained by linear interpolation of the differential phase-spectrum to $k=9.83038$. **(f–k)** Use of the sDFT with frequency-domain windowing to determine the phase difference between two mono-sinusoidal signals. (**f**) Simulated 12-bit reference (red) and object (blue) signals, with a phase difference of $-\pi /3$. (**g**) DFT magnitude spectra of input signals, calculated using the sDFT. (**h**) DFT phase-spectra of input signals, calculated using the sDFT. (**i**) DFT magnitude spectra of input signals after frequency-domain windowing. The vertical line at $k=9.83038$ denotes the signal frequency extracted from the windowed DFT using Eq. (8). (**j**) DFT phase-spectra of input signals after frequency-domain windowing. (**k**) Differential phase-spectrum $\Delta \phi ={\phi }_{{\rm{obj}}}-{\phi }_{{\rm{ref}}}$, wrapped to lie in the range $-\pi  < \Delta \phi \le \pi$. The phase difference $\Delta \phi =-\,1.04711$ is obtained by linear interpolation of the differential phase-spectrum to $k=9.83038$.

The use of the above procedure to calculate the phase difference between two signals is illustrated in Fig. 2, using (simulated) digitised sinusoids with a common time period of 0.0625 s, a constant phase difference $\Delta \phi ={\phi }_{{\rm{obj}}}-{\phi }_{{\rm{ref}}}=\pi /3$ and a signal-to-noise ratio of 1000 (see Fig. 2a). The 1024-sample signals have been digitised at 12-bit resolution over the range 0 to 1 with a time interval of 0.6 ms per sample, similar to the settings used for the experimental measurements reported below. In Step 1, we multiply the two signals by the Hann window function, yielding two tapered signals that begin and end at approximately zero (Fig. 2b). In Step 2, we take Fast Fourier Transforms of the windowed signals, yielding the magnitude and phase spectra shown in Figs. 2c,d. The two magnitude spectra are virtually identical (as expected), each showing a single peak centred at $k=10$. In Step 3, we substitute ${k}^{\ast }=10$ into Eq. (8), yielding interpolated $\hat{k}$-values of 9.8304058 and 9.8303621for the two signals. These are then averaged to obtain a mean $\hat{k}$-value of 9.8303839 which, remembering ${f}_{k}=k{f}_{s}/N$, corresponds to a frequency of 15.999974 Hz (compared to a ‘real’ frequency of 1/0.0625 = 16 Hz). The two phase spectra are offset relative to one another (as expected), and each shows a “blip” or phase-flip about $\hat{k}$. To eliminate the phase-flips (and hence permit a more reliable interpolation of the phase), in Step 4 we subtract one phase spectrum from the other to obtain a differential phase spectrum, wrapping the result to lie in the range $-\pi$ to $+\pi$ if necessary (Fig. 2e). Finally in Step 5, we linearly interpolate the differential phase spectrum to $\hat{k}$ to obtain an estimate of −1.0471100 rad for the phase difference between the signals. This compares to the ‘true’ phase difference of −$\pi /3=-1.0471976$ rad – a difference of 8.7548e × 10^−5^ rad or 0.005°. (Note, repeating the measurement would yield a slightly different value for the phase difference since the result is affected by signal noise. The phase difference should therefore be averaged over multiple measurements to obtain a reliable estimate of the phase, see Supplementary Fig. S5).

Note, for clarity we have omitted bins 0 to 2 which have their origin in the DC values of the two signals. (Substituting $k=0$ into Eq. (7), yields $Y(0)=\mathop{\sum }\limits_{n=0}^{N-1}y(n)$, so the first bin is proportional to the mean of the input signal. The combined effects of spectral leakage and windowing ‘blur’ the DC signal into the adjacent bins).

### Background to the sliding DFT

The above analysis procedure requires just one DFT to analyse each signal, substantially reducing the computational effort compared to the original method of Vishnyakov *et al*. However, calculating even a single DFT via the FFT algorithm may be too demanding for real-time operation on a microcontroller. To further reduce the computational demand, we therefore switch from the FFT to the sDFT^13,14,19^, which updates the DFT on a point-by-point basis and so spreads the workload uniformly across the full data acquisition cycle.

The sDFT is based on a sliding window of *N* data points, which advances by one point every time a new sample is acquired (with the oldest data point dropping out of the window), see Supplementary Appendix S1. Here we use a stabilised version of the sDFT given by Eq. (9)^20,21^:

9 $${Y}_{m+1}(k)=r{e}^{i2\pi k/N}[{Y}_{m}(k)-{r}^{N}y(m)+y(m+N)]$$

where *r* is a constant that is very slightly smaller than one and $m\Delta t$ is the time of the first sample in the current window. Assuming for a moment that $r=1$, it can be seen that to advance the *k*-th bin of the DFT by one point in time, we simply add the newest data point $y(m+N)$ to the current bin value ${Y}_{m}(k)$, subtract the oldest data point $y(m)$, and then multiply the total by the phase factor ${e}^{i2\pi k/N}$. (The reason for using an *r*-value slightly smaller than one is to counter the effect of rounding errors in the calculation of the ${e}^{i2\pi k/N}$ pre-factor, which can lead to complex magnitudes slightly greater than one and so cause the output to become unstable^20^). The sDFT uses two arrays of length *N*, which are separately used to store the *N* most recent *y*-values of the input data and the (constantly updated) *Y*-values of the DFT. The array contents are initially set to zero, and Eq. (9) is then applied to each frequency bin every time a new data point is acquired. The output of the sDFT therefore becomes valid as soon as the *N*-th data point is acquired, and remains valid from that point onwards.

There are two important features of the sliding DFT that make it especially appealing for real-time applications. Firstly, the computational effort is spread evenly over the *N*-samples of the DFT window, which means there is no need to pause for calculation after data acquisition is completed. Secondly, since each bin is calculated independently of the other bins, one may choose to calculate only those frequency bins that are of interest. In the current context, where we are dealing with mono-sinusoidal signals, this latter property leads to a massive reduction in computational complexity, allowing us to focus on a small cluster of frequency bins around the fundamental frequency while ignoring all the others.

A minor drawback of the sDFT is that it does not readily permit the use of time-domain windowing, since multiplying by the window function would spoil the computational simplicity of Eq. (9). Fortunately, exploiting the fact that multiplication in the time domain is equivalent to convolution in the frequency domain, windowing may be achieved by filtering the calculated DFT signal. The Hann function discussed above is especially straightforward to apply in the frequency domain, with the *k*-th bin ${\tilde{Y}}_{m}(k)$ of the windowed DFT spectrum being given by^16,17^:

10 $${\tilde{Y}}_{m}(k)=-\,\frac{1}{4}{Y}_{m}(k-1)+\frac{1}{2}{Y}_{m}(k)-\frac{1}{4}{Y}_{m}(k+1)$$

which is a simple weighted sum of the *k*-th bin (of the unwindowed data) and its two immediate neighbours.

The modified procedure (Method 2) for calculating the phase difference via the sDFT is shown in the right-hand column of Fig. 2. Figure 2f shows the same input signals as Fig. 2a. In Step 1, we calculate the discrete Fourier transform of the input data using the sDFT algorithm, which yields the magnitude and phase spectra shown in Fig. 2g,h, respectively. Owing to the effects of spectral leakage, the peaks are broader (i.e. decay to zero more slowly) than the windowed data in Fig. 2c and the magnitude and phase spectra are slightly different in shape for the two signals (i.e. the red circles of the reference spectrum do not sit exactly on top of the blue lines of the object spectrum). In Step 2, we apply Eq. (10) to the two DFTs in order to implement windowing in the frequency domain, yielding the magnitude and phase spectra shown in Fig. 2i,j, which are virtually identical to those obtained by Method 1. In Step 3, we apply Eq. (8) to the filtered ${\tilde{Y}}_{m}(k)$ values, which yields an average $\hat{k}$ value of 9.8303839, virtually identical to that obtained by Method 1. (The values differ in the sixteenth significant figure). In Steps 4 and 5, we calculate the differential phase spectrum (Fig. 2k) and interpolate to $\hat{k}$, obtaining an estimated phase difference $\Delta \phi =-\,1.0471100$, which again corresponds closely with the value obtained by Method 1. (The values differ in the fifteenth significant figure).

As the sDFT window moves forward in time, the calculated phase difference fluctuates about the ‘true’ phase due to noise and rounding errors caused by the 12-bit digitisation, see Supplementary Fig. S5. Hence, to obtain a reliable, unbiased estimate of the true phase difference, the calculated phase difference should be averaged over an extended time period equal to several windows of the sDFT. For noise levels > 0.2% the fluctuations are almost entirely attributable to noise, and 12-bit digitisation gives equivalent results to 16- and 32-bit digitisation (see Supplementary Fig. S5e–j). Hence, the 13-bit ADCs on the Teensy 3.6 may be safely used for data acquisition without compromising performance, beneficially avoiding the need for external data acquisition hardware.

### Experimental implementation

The motor is driven at a fixed speed of approximately eight revolutions per second, using a dedicated electronic speed controller. (The exact speed is not important). To carry out the phase calculations we set the window size to 1024 samples and use three frequency bins either side of the strongest frequency bin in the DFT magnitude spectrum. Hence, it is necessary to calculate just seven of the 1024 frequency bins in the full DFT spectrum, resulting in a substantial reduction in computational effort compared to the FFT. The calculations are carried out in 32-bit precision using the onboard 32-bit Floating Point Unit (FPU) of the Teensy 3.6 microcontroller, which provides an approximate thirty-fold speed-up relative to software-based calculations at the same precision. The Teensy 3.6 has two built-in ADCs, which permit simultaneous measurement of the outputs from the two amplified photodiodes. The ADCs are operated with a sample time of 0.6 ms (*f*_*s*_ ≈ 16.67 kHz) at 12-bit resolution, which offers an acceptable trade-off between time resolution and noise level. To compensate for drift, the laser intensity is monitored using a light to frequency converter connected to a digital input pin of the microcontroller, and the measured laser intensity is smoothed using an exponential filter^22^ with time constant *τ* = 0.06 s. Both ADC signals are divided by the filtered intensity prior to signal processing. The phase difference is updated every sample using the sDFT-based algorithm and smoothed by an exponential filter (see below). Every 64th value of the smoothed phase difference is sent to a remote PC (or other device) for data visualisation via the USB interface of the Teensy 3.6.

## Results

**Figure 3:**
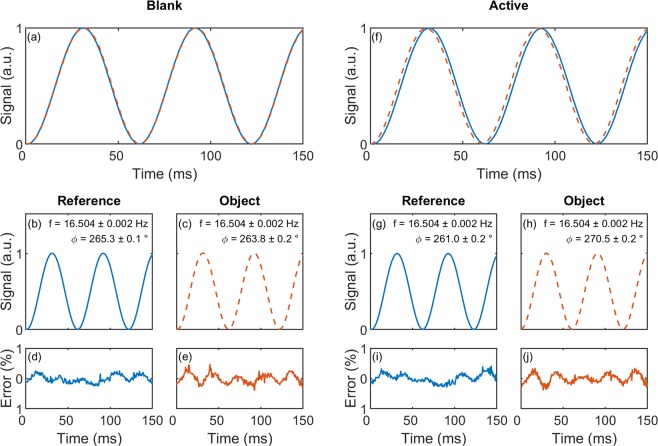
(**a**) Normalised experimental input signals for a typical “blank” measurement, using ultra-pure water and a 5-cm optical cell. The reference and object signals are denoted by a blue solid line and an orange dashed line, respectively. The signals were obtained using a 0.6-ms sample time and a motor speed of approximately eight revolutions per second. **(b,c)** Least-squares optimised mono-sinusoidal fits to the reference and object signals. **(d,e)** Errors in the sinusoidal fits, expressed as a percentage of the mean signal value. **(f–j)** Equivalent plots for an “active” measurement, with a 202 mg/ml sucrose solution in the optical cell.

Figure 3a–e illustrate the behaviour of the polarimeter during a blank measurement with ultra-pure water in the 5-cm optical cell. Figure 3a shows scaled and normalised optical signals measured by the two amplified photodiodes. The object and reference signals overlap closely since the polarisation axes of P1 and P2 are closely aligned and water is optically inactive. Figure 3b,c show least squares nonlinear fits of the two signals to mono-sinusoids, while Fig. 3d,e show the corresponding residuals. The fitted sinusoids have similar frequencies of $16.504\pm 0.002\,{\rm{Hz}}$ – twice the rotational frequency – with a phase difference $\Delta \phi ={\phi }_{{\rm{obj}}}-{\phi }_{{\rm{ref}}}$ of −1.5 ± 0.3° (99% confidence intervals). The root-mean squared error of both fits is 0.0024 compared to a signal amplitude of one, indicating the signals are accurately described by pure mono-sinusoids in agreement with Eqs. 3a and 3b.

Figure 3f–j show equivalent data for an active measurement, using an enantiomerically pure 202 mg/ml solution of D-sucrose molecules in water. The signals are again well described by pure mono-sinusoids of frequency 16.504 ± 0.002 Hz with similar root mean squared errors of 0.0029. The effect of the optically active sucrose molecules is to induce a positive optical rotation in the plane of polarisation of the object beam, which manifests itself as a large positive phase-difference of 9.5 ± 0.4° between the object and reference beams. Hence, applying Eq. (6), we obtain an optical rotation angle of 0.5 × (9.5−(−1.5)) = 5.5 ± 0.4°.

**Figure 4:**
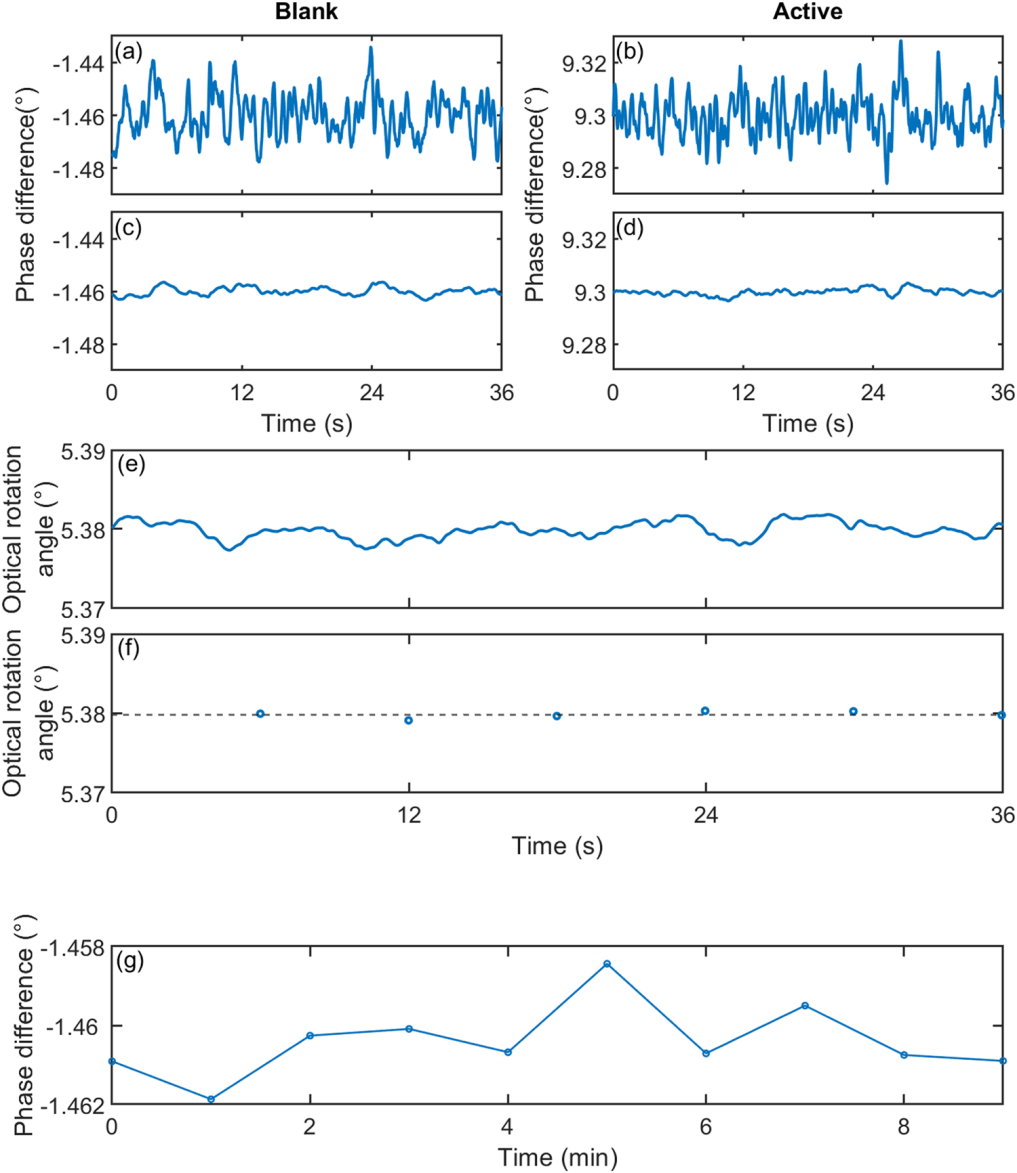
(**a**,**c**) Time series showing the experimentally determined phase-difference between the object and reference signals for a typical “blank” measurement, before (**a**) and after (**c**) exponential filtering. The measurements were obtained with ultra-pure water using a 5-cm optical cell, with a 0.6-ms sample time, an sDFT window size of 1024 data points, and a motor speed of eight revolutions per minute. Exponential filtering was carried out using *λ* = 0.0128, equivalent to a 2.9-s time constant, see Eq. (11). **(b,d)** Time series showing the experimentally determined phase-difference between the object and reference signals for a typical “active” measurement, before (**b**) and after (**d**) exponential filtering. The data were obtained with 202 mg/ml sucrose in the optical cell, using the same settings as for the blank measurement. **(e)** Time series showing the optical rotation angle calculated from the data in (**c**,**d**), using Eq. (6). **(f)** Down-sampled time series for the optical rotation angle, obtained by averaging the data in (**e**) in 6-s blocks. The dotted line represents the mean optical rotation angle of 5.3798°. The standard deviation is $0.00044$, implying a $3\sigma$ accuracy of ±0.0013 **(g)** Results of ten successive measurements of the phase difference between the object and reference signals for a typical blank measurement, obtained with ultra-pure water in the 5-cm optical cell. The measurements were recorded at 1-minute intervals, using a 6-s time average of the exponentially filtered phase and identical settings to those used in (**e**). The measurements are distributed about a mean value of −1.4604° with a standard deviation of 0.00093°, implying a $3\sigma$ precision of ±0.0028°. The straight lines between points are a guide to the eye.

Using nonlinear regression to analyse the data is too computationally intensive for real-time application on a microcontroller, and results in a substantial uncertainty in the rotation angle of ±0.4°. The sDFT by contrast offers an efficient and accurate method for extracting the phase difference between the two signals that may be readily implemented on embedded hardware. Figure 4a,b show time series for the sDFT-derived phase differences with ultra-pure water and 202 mg/ml D-sucrose in the optical cell, using Δ*t* = 0.6 ms, *N* = 1024 and 12-bit digitisation as before. The microcontroller carries out the sDFT calculations in the intervals between samples, meaning the phase calculation is updated every 0.6 ms. However, to keep the streaming rate to a manageable level, only one value in sixty-four is outputted over the USB interface of the microcontroller. The time interval between data points in Fig. 4a,b is therefore $0.6\times 64=38.4$ ms. The time series begin with the data arrays initialised to zero, and the output therefore becomes valid $0.6\times 1024=\,614.4$ ms after starting the measurement. Ignoring the initialisation phase, the blank and active phase differences have mean values of −1.4601° and 9.2995°, respectively. However, owing to measurement noise, significant fluctuations are evident in the reported phase, similar to the simulated signals in Supplementary Fig. S5.

To smooth out these fluctuations, we apply an exponential filter of the form

11 $$\Delta \varPhi ({t}_{i})=[1-\lambda ]\Delta \varPhi ({t}_{i-1})+\lambda \Delta \phi ({t}_{i})$$

where $\Delta \phi ({t}_{i})$ is the unfiltered phase difference, $\Delta \varPhi ({t}_{i})$ is the filtered phase difference, and *λ* is a smoothing factor $(0\le \lambda \le 1)$^22^. Figure 4c,d show the filtered phase-difference versus time using a $\lambda$-value of 0.0128, which corresponds to a time constant *τ* of 2.9 s – approximately five DFT window lengths. (The plots again show every 64th value of the internally calculated filtered phase-difference). The exponential filter suppresses the high frequency fluctuations, yielding slow-varying output values that move around mean values of −1.4601° and 9.2996° for the blank and active phase differences, virtually the same values as for the unfiltered signals. Using ${\theta }_{{\rm{rot}}}({t}_{i})=0.5[\Delta {\varPhi }_{{\rm{obj}}}({t}_{i})-\Delta {\varPhi }_{{\rm{ref}}}({t}_{i})]$ as a filtered version of Eq. (6), we therefore obtain a mean rotation angle ${\bar{\theta }}_{{\rm{rot}}}$ of 5.3798° with a standard deviation of 0.0010 (see Fig. 4e). This is consistent with the value of $5.5\pm 0.4^\circ$ obtained by least-squares fitting, but with a substantially reduced uncertainty.

The accuracy of the measured optical rotation angle depends on the total measurement time. For instance, averaging the filtered output of Fig. 4e over a period of 6 s yields the down-sampled signal shown in Fig. 4f with a standard deviation of 0.00044. Defining the measurement accuracy as the $3\sigma$ uncertainty in the down-sampled signal, this corresponds to an accuracy of ±0.0013. The trade-off between measurement time and accuracy is shown in Supplementary Fig. S6, with longer averaging times yielding better accuracies as expected. (Measurement times of several seconds are typical for commercial instruments operating in high resolution mode, see Supplementary Table S2). Using a 6-s measurement time, we also determined the precision (reproducibility) of the polarimeter by carrying out ten 6-s blank measurements at one-minute intervals, a sufficient separation in time to ensure no data overlap between successive measurements (see Fig. 4g). The mean phase difference was −1.4604° with a standard deviation of 0.00093°, implying a $3\sigma$ precision of ±0.0028°. Hence, it follows from Eq. (6) that the precision in the optical rotation angle is also ±0.0028°. To place these values in context, the Vishnyakov polarimeter was quoted as having a $3\sigma$ accuracy of ±0.0014°, while high-resolution commercial polarimeters from leading manufacturers have typical accuracies and precisions of ±0.002° (see Supplementary Table S2). Hence, despite its low cost, the polarimeter described here is a research-grade instrument with close to state-of-the-art accuracy and precision.

**Figure 5:**
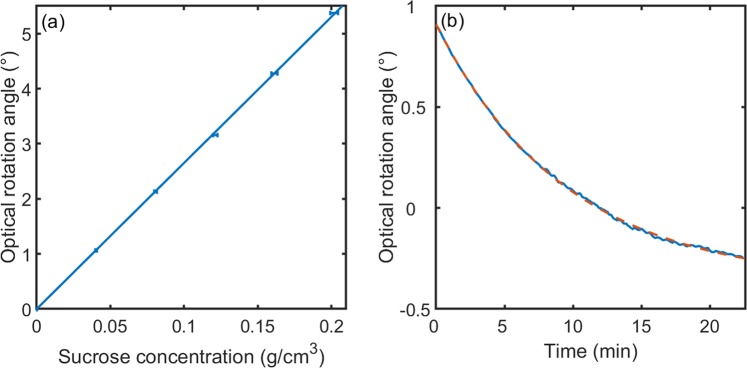
**(a)** Plot of measured optical rotation angle vs sucrose concentration, obtained using a 5-cm optical cell. The measurements were carried out using the same settings as for Fig. 4, except for a slightly reduced averaging time of 3.84 s (equal to 100 outputs of the microcontroller). The markers denote experimental data, while the solid line shows a least-squares optimised linear fit to ${\theta }_{{\rm{rot}}}=l{\theta }_{{\rm{s}}}[S]$, with *R*^2^ = 99.96% and *θ*_s_ = 53.0 ± 0.2 deg·mL·g^−1^·dm^−1^ (20 °C, 650 nm). **(b)** Plot of measured optical rotation angle versus time, obtained using a 0.2 g/ml sucrose solution in 1.34 M HCl and a 1-cm path-length cuvette (blue line). The measurements were carried out using the same settings as for Fig. 4. Hydrolysis of dextro-rotatory sucrose to a laevo-rotatory mixture of glucose and fructose induces an exponential decrease in the optical rotation angle from 0.8975° at *t* = 0 min to −0.2445° at *t* = 22.4 min. The dashed orange line shows a least squares curve fit to a mono-exponential decay, with a decay constant of (1.745 ± 0.002) × 10^−3^ s^−1^.

By carrying out phase-difference measurements for a six-membered linear concentration series spanning the range 0 to 0.202 g/ml, we used the polarimeter to determine the specific optical rotation ${\theta }_{{\rm{s}}}$ of D-sucrose. The solutions were tested in a randomised order, rinsing thoroughly with ultra-pure water between each test. For each concentration the rotation angle was determined by averaging 100 outputs of the microcontroller over a 3.84 s time period. Figure 5a shows the dependence of the rotation angle on the sucrose concentration [S]. The rotation angle ${\theta }_{{\rm{rot}}}$ is theoretically related to [S] by the linear equation ${\theta }_{{\rm{rot}}}=l{\theta }_{{\rm{s}}}[S]$, where $l=5$ cm is the path-length of the optical cell. Least-squares fitting yields a ${\theta }_{{\rm{s}}}$ value of 53.0 ± 0.2 deg·mL·g^−1^·dm^−1^ and a high linear correlation coefficient of 99.96%. This is consistent with a value of 54° ± 2° calculated using the Drude model ${\theta }_{s}=\,A/({\lambda }^{2}-{\lambda }_{0}^{2}\,)$ with *A* = 2.16 × 10^7^ nm^2^dm^−1^g^−1^mL and *λ*_0_ = 146 nm as previously reported by Compton *et al*. for D-sucrose in water (20 °C, 650 nm)^23^. In Supplementary Fig. S7, we show (for a 100 mg/ml sucrose solution) how the measured value of the optical rotation angle is affected by the presence of a strongly absorbing (optically inactive) contaminant, and find the polarimeter is able to handle absorbances of up to one without a significant deterioration in performance. For higher absorbances, the neutral density filter ND should be removed and/or a stronger laser should be used to maintain accuracy.

Finally, we describe the use of the polarimeter to monitor the acid-catalysed hydrolysis of dextro-rotatory sucrose to a laevo-rotatory mixture of fructose and glucose. Figure 5b shows the time evolution of the optical rotation angle for 0.2 g/ml sucrose in 1.34 M HCl, using a 1-cm path-length cuvette. The extracted rotation angle varies smoothly with time, albeit with some minor fluctuations starting at *t* = 7 min due to bubble formation within the cuvette. The reaction kinetics are well described by a single exponential function of the form ${\theta }_{{\rm{rot}}}(t)=\,{\theta }_{\infty }+\,A{e}^{-kt}$ with values of −0.37°, 1.28° and 0.00175 s^−1^ for ${\theta }_{\infty }$, *A* and *k* respectively.

An extracted reaction rate constant of 0.104 ± 0.008 min^−1^ is in agreement with literature reports^24^. Another application of the polarimeter, namely the determination of the lactose content of a milk sample, is described in Supplementary Fig. S8.

## Conclusion

In conclusion, we have developed an affordable, high performance laser polarimeter based on a design by Vishnyakov *et al*. The instrument uses separate reference and object beams derived from a common laser source. The reference beam passes through a fixed polariser and a rotating polariser, while the object beam passes through another fixed polariser, an optical cell, and the same rotating polariser. The rotating polariser induces a sinusoidal variation in the transmittances of the two beams, with the phase difference between the two beam intensities depending on the relative orientation of the fixed polarisers and the optical activity of the sample under test. The rotation angle is determined by two successive measurements of the phase difference, with and without an optically active sample in the path of the object beam.

In contrast to null-point methods, the chosen phase-based method does not require high-precision goniometric devices or servo actuators, and may be constructed using a small number of readily available off-the-shelf optical and mechanical parts. By replacing a number of costly components in the Vishnyakov design by cheaper alternatives, we have reduced the bill-of-materials cost to <£300, without compromising instrumental performance. The principal changes are the replacement of a HeNe laser by a 650 nm solid-state laser diode; the replacement of a prism beam-splitter by a plate beam-splitter; the replacement of Glan-Thomson prism polarisers by thin-film polarisers; and the replacement of a PC equipped with a 24-bit data acquisition card by a low-cost microcontroller. The latter substitution was made possible by our use of a streamlined data analysis procedure based on the sliding discrete Fourier transform, which greatly reduces the computational burden involved in calculating the phase differences.

The polarimeter is a fully automatic research-grade instrument that calculates the optical rotation *at the push of a button*, with an accuracy of ±0.0013° and a precision of ±0.0028° – comparable to far costlier commercial instruments. For illustrative purposes, we have applied it here to the determination of the specific rotation angle of D-sucrose in water, obtaining a value of 53.0 ± 0.2 deg·mL·g^−1^·dm^−1^ at 650 nm and 20 °C, in agreement with literature reports. We also demonstrated its suitability for studying the kinetics of chiral reactions, exploiting the real-time nature of the sDFT algorithm to allow uninterrupted reaction monitoring. The polarimeter’s ease of use, compact size, fast measurement time and high angular resolution make it a capable and versatile tool for analytical science, while its low cost means it is ideally suited for use in resource-constrained environments and process monitoring. The polarimeter is released here as open hardware, with technical diagrams, a full parts list, and source code for its firmware included as Supplementary Information.

## Supplementary information

Supplementary Information.
