# Supplementary material for: A high-resolution polarimeter formed from inexpensive optical parts
Source: Sci Rep. 2020 Mar 25;10:5448. doi: 10.1038/s41598-020-61715-7 (PMC7096414; doi:10.1038/s41598-020-61715-7)
Supplement: Supplementary file 1 — Supplementary Information. [file 41598_2020_61715_MOESM1_ESM.pdf]

A high-resolution polarimeter formed from inexpensive optical parts

A. J. Harvie[1], T. W. Phillips[2] and J. C. deMello[1*]

[1] Department of Chemistry, NTNU, Trondheim, Norway

[2] Department of Chemistry, Imperial College London, United Kingdom

*john.demello@ntnu.no

Supplementary Information

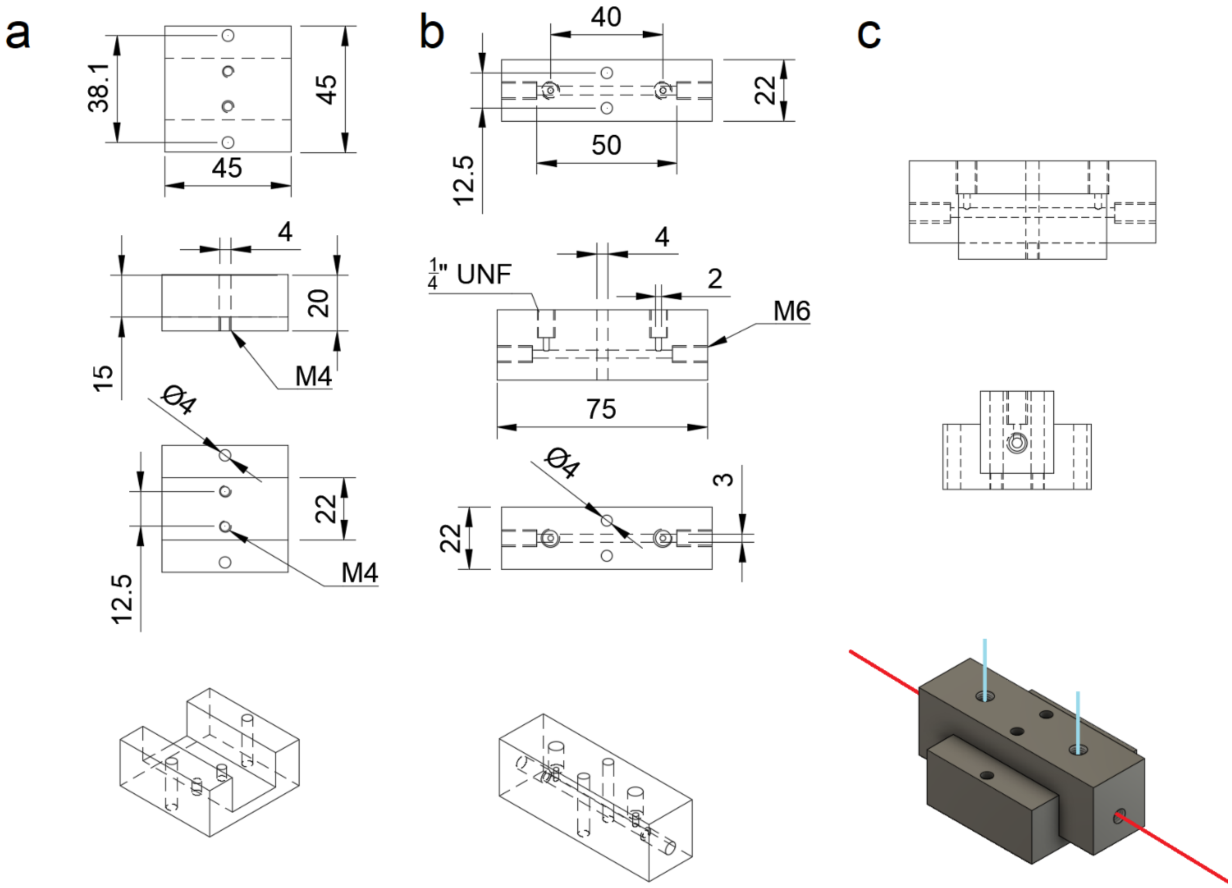

Fig. S1 Technical diagrams showing construction of a 50-mm optical cell (dimensions in millimetres). The cell is fabricated from two interlocking milled-out blocks of black PTFE: a mounting block (a) and an optical block (b). A horizontal measurement channel is formed in the optical block by drilling a 3-mm hole through the length of the block. 5-mm-diameter ports of length 12.5 mm are drilled at each end of the through-hole, and tapped with M6 threads. Two vertical holes for the fluidic inlet and outlet (blue lines) are drilled from the top of the block to the through-hole and tapped with ¼" UNF fittings. 5-mm glass discs are inserted into each port and held firmly against the measurement channel using hollow-core M6 screws, providing a clear, sealed optical path through the cell (red line). The assembled block is shown in (c).

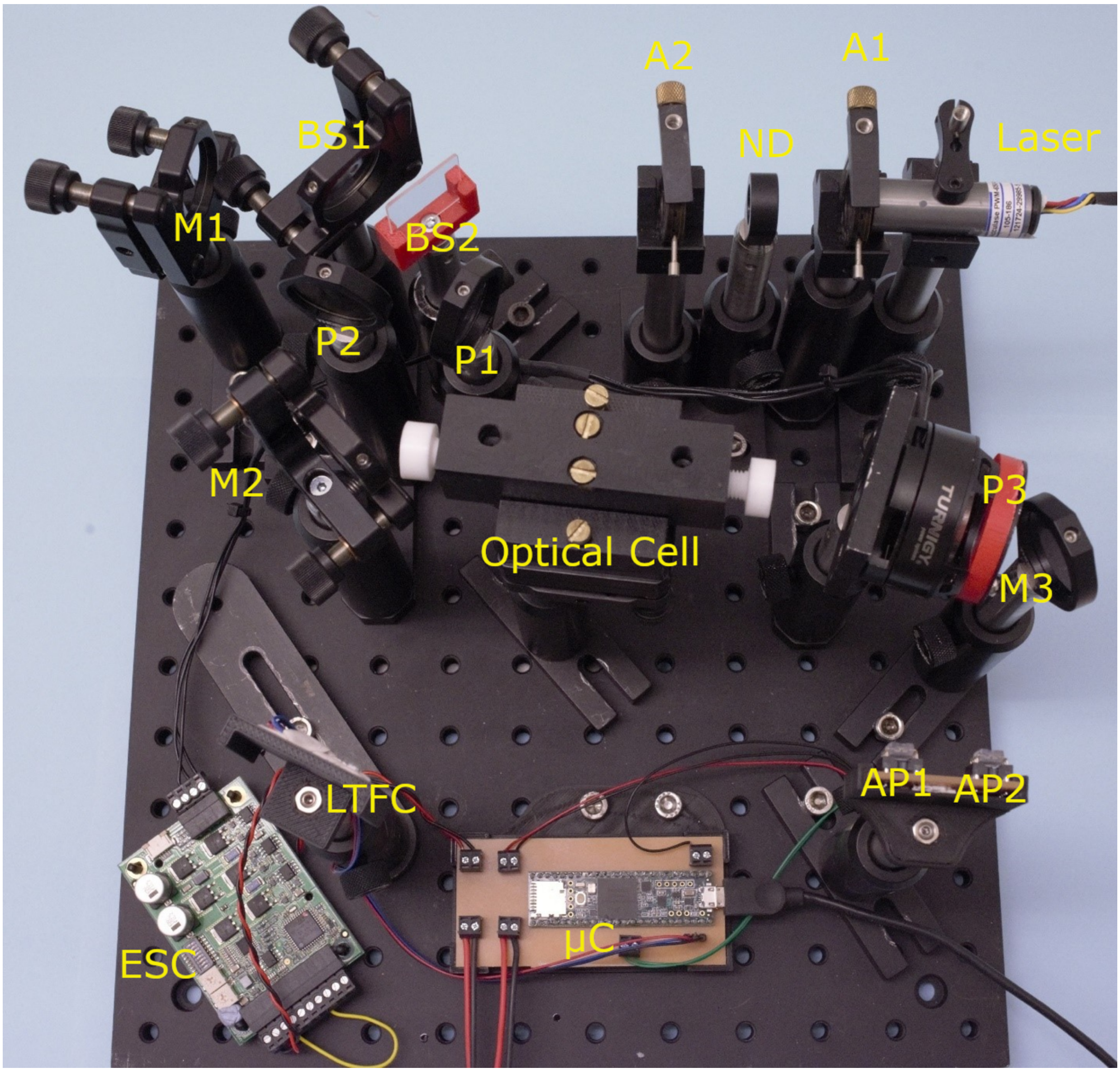

Fig. S2 Photograph of the assembled polarimeter. The beam from the laser is passed through a 1-mm aperture (A1), a neutral density filter (ND) and a second 1-mm aperture (A2). A 50:50 plate-type beam-splitter (BS1) divides the laser beam into an object beam and a reference beam. The reference beam is directed by a plane mirror (M1) through a fixed thin-film polariser (P1) onto the centre of a rotatable thin-film polariser (P3), which is mounted on a hollow-shaft motor. The motor is driven at a continuous speed of approximately eight revolutions per minute by an electronic speed controller (ESC). The object beam is directed by BS1 through a fixed thin-film polariser (P2), and onto plane mirror (M2); it then passes through a 5-cm optical cell, before striking the same central point on the rotating polariser (P3). The object and reference beams pass through the centre of P3 and are then directed by a third mirror (M3) onto a pair of amplified photodiodes (AP1, AP2). The beam splitter BS2 is a glass slide, which directs a small fraction (~ 4 %) of the initial beam onto a light-to-frequency converter (LTFC). The polarimeter components are mounted on a 12”×12” breadboard using standard optical mounts. The microcontroller (μC) is soldered to a custom printed circuit board (PCB) which provides electrical connections to the optical sensors and power connections for the motor. The two amplified photodiodes are mounted on a separate PCB. The two PCBs sit in custom 3D-printed mounts.

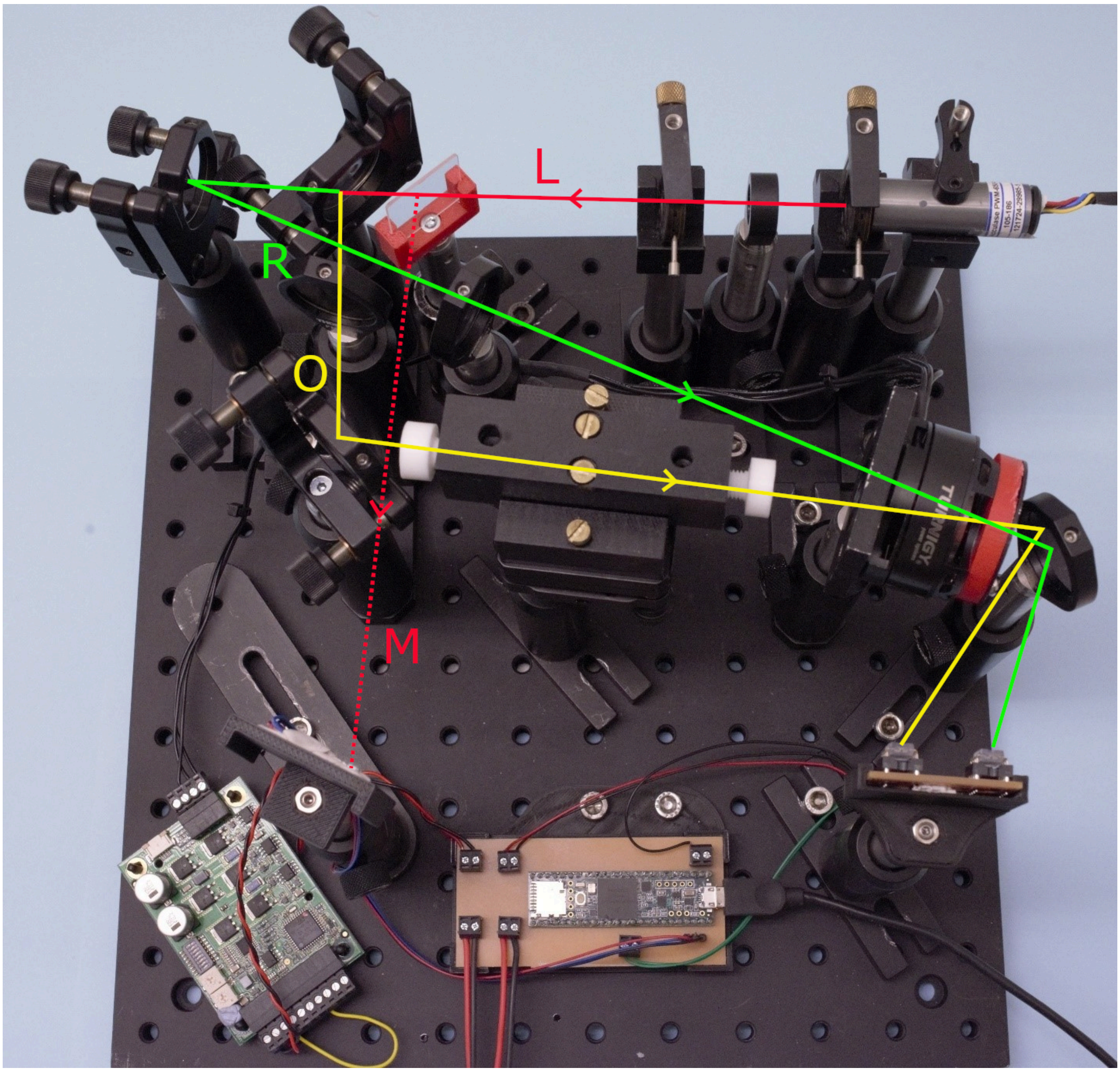

Fig. S3 Annotated photograph of the assembled polarimeter, showing optical beam paths. The initial laser beam (L) strikes a beam-splitter formed from a glass slide. The (weak) reflected beam acts as a low-intensity "monitor beam" (M), allowing variations in laser intensity to be observed using a light-to-frequency converter. The transmitted beam strikes a 50:50 plate beam-splitter, where it is divided into an object beam (O) and a reference beam (R). See Fig. S2 for further details.

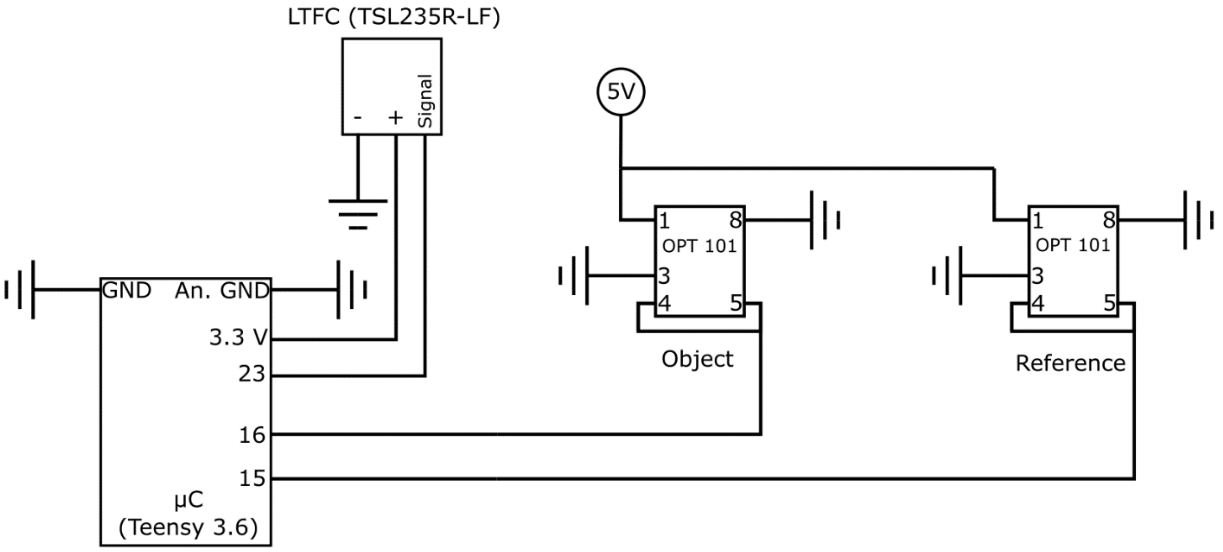

Fig. S4 Circuit diagram of detection circuitry, comprising one microcontroller development board (Teensy 3.6, PJRC), two amplified photodiodes (OPT 101, Texas Instruments) and a light-to-frequency converter (TSL235R-LF, AMS). The analogue signals from the amplified photodiodes are measured using the microcontroller's two built-in analogue to digital converters, while the digital signal from the light-to-frequency converter is read using one of its digital I/O pins. The object and reference signals are divided by the measured laser intensity prior to calculating the phase difference. The calculated phase difference is sent via the microcontroller's USB port to a PC or other remote device for data visualisation.

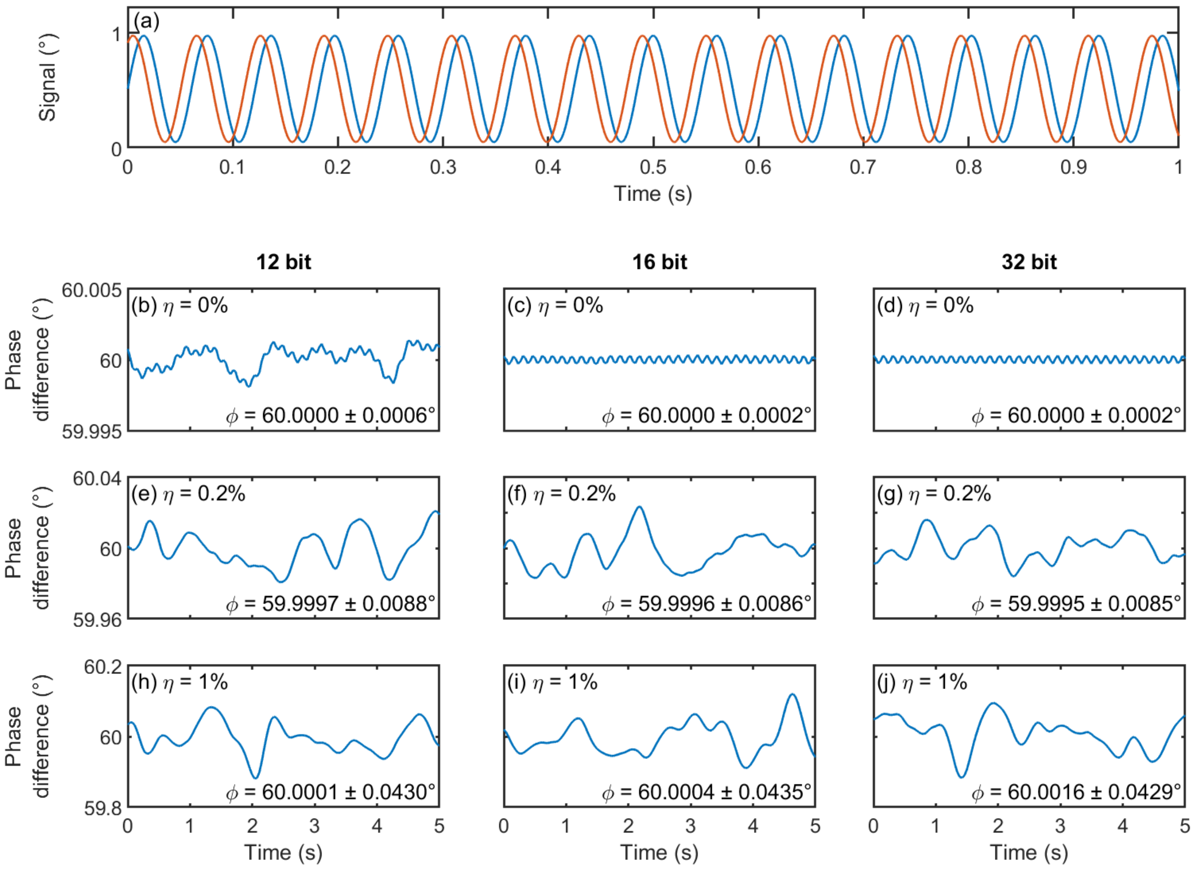

Fig. S5 Simulations showing the effect of ADC-bit-depth and signal noise on the calculated phase difference. **(a)** 16.5 Hz input signals with a 60° phase difference between the object (orange line) and reference (blue line) signals. **(b-j)** Calculated phase difference versus time for different bit-depths and Gaussian noise levels (η), using a sample time of 0.6 ms and a sDFT window size of 1024 data points.

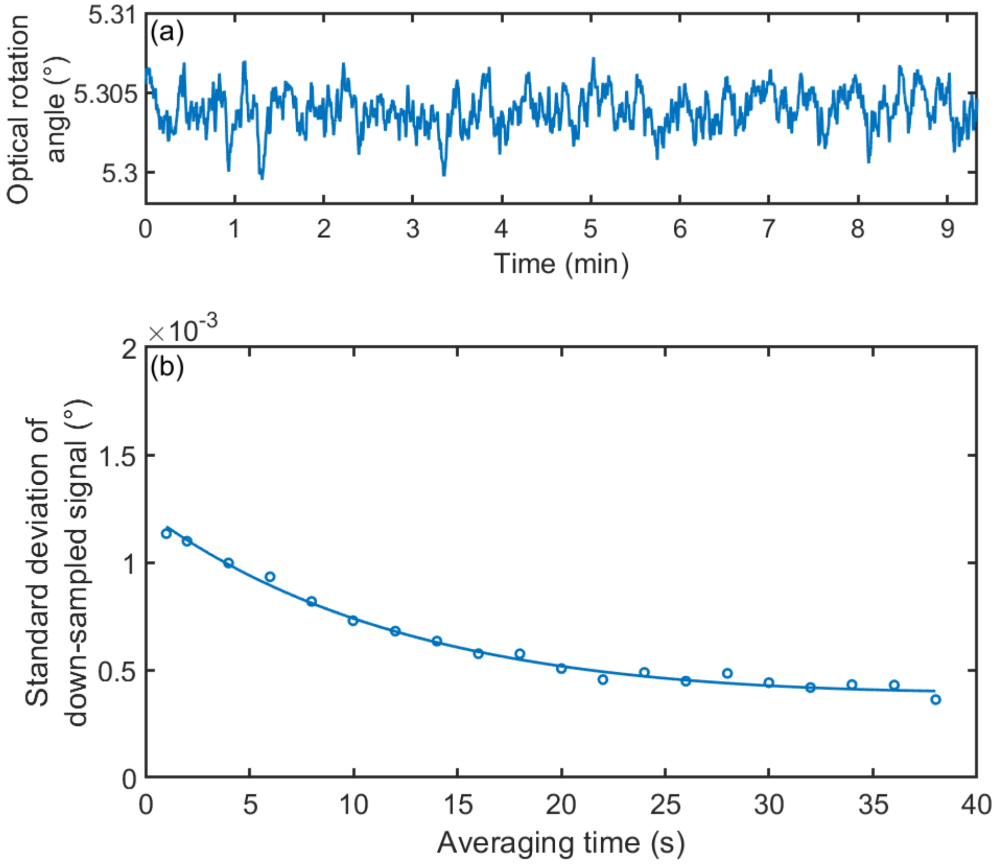

Fig. S6 **(a)** Calculated optical rotation angle versus time, using ultra-pure water and 200 mg/ml sucrose in water for the blank and active measurements, respectively (5-cm optical cell). The data were obtained using a sample time of 0.6 ms, a sDFT window size of 1024 data points, and exponential filtering with $\tau = 2.9$ s. **(b)** Standard deviation of down-sampled signal versus averaging time, indicating a progressive drop in the standard deviation with increasing averaging time. The solid curve is a guide to the eye.

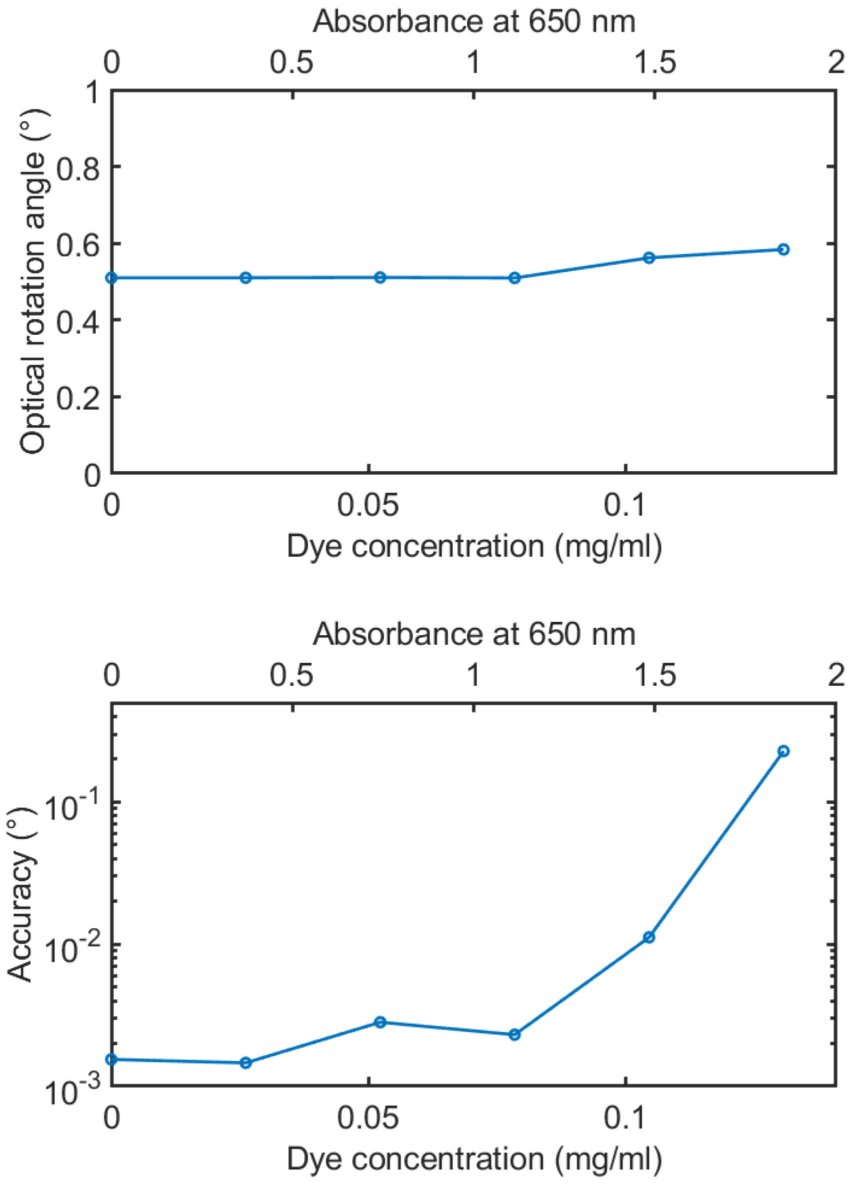

Fig. S7 Plot showing the mean measured optical rotation angle and accuracy for 100 mg/ml sucrose in the presence of varying concentrations of the (optically inactive) blue dye xylene cyanol FF, using a 1-cm pathlength cuvette. The secondary *x*-axis shows the corresponding optical density at the 650-nm probe wavelength. The accuracy is better than 0.003º for optical densities of 1.1 or less, increasing rapidly above this value due to inadequate use of the dynamic range of the analogue to digital converters (ADCs) on the microcontroller. To maintain accuracy at optical densities substantially greater than one, the neutral density filter ND should be removed from the optical set up and/or a stronger laser should be used.

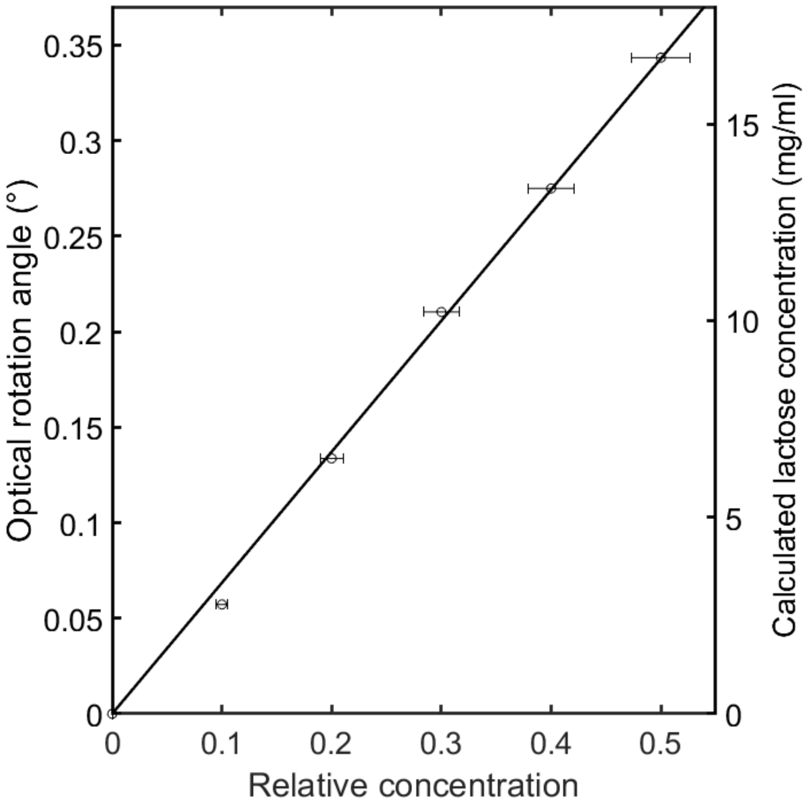

Fig. S8 Use of polarimeter to determine the lactose concentration in whole milk. The plot shows the measured optical rotation angle at 650 nm versus the volumetric concentration of deproteinated whole milk, using a 5-cm optical cell. The markers denote experimental data, while the solid line shows a least-squares optimised linear fit to $\theta_{\rm rot} = l\theta_{\rm s}[S]$, with R^2 = 99.81 %. The secondary y-axis indicates the calculated lactose concentration corresponding to each measured optical rotation angle, assuming a specific optical rotation of 41 ± 12 deg·mL·g^{-1}·dm^{-1} at 650 nm. Extrapolating to 100 % volumetric concentration yields a lactose concentration of 33 ± 10 mg/ml lactose for the original milk sample in agreement with literature values [1].

Experimental procedure. A whole milk sample was obtained from Tinemelk SA (Tinemelk Helmelk, 3.5 % fat content). Following Ref. 2, the milk solution was deproteinated by adding 7.5 ml of concentrated H_2SO_4 to 50 ml of whole milk, followed by 7.5 ml of 1M aqueous potassium iodide to induce precipitation of milk fats and proteins. The product was filtered, and the collected filtrate was diluted in water to a volume of 100 ml, creating a colourless (non-scattering) stock solution with half the volumetric concentration of the original sample. The stock solution was used to prepare solutions with volumetric concentrations in the range ten to fifty percent of the original sample, i.e. with relative concentrations of 0.1 to 0.5. The specific angle of rotation at 650 nm was calculated using the Drude model $\theta_s = A/(\lambda^2 - \lambda_0^2)$, with A = 1.47 ± 0.70 nm^{2}dm^{-1}g^{-1}mL and λ_0= 254 ± 70 nm, see Ref. 3.

[1] R. M. Feeley, P.E. Criner, H.T. Slover, Journal of the American Dietetic Association 1975, 66, 2.

[2] R. Scott, J.E. Scott, R. K. Robinson, R. A. Wilbey. Cheesemaking practice, Springer 1998.

[3] K Atroshchenko et al. Journal of Physics: Conference Series 2012, 398, 1.

Part	Approx. Cost
Laser diode (Acculase PWM-650-1-S, Global Laser)	£145 (cheaper options available)
Plate beam-splitter (EBS1, Thorlabs)	£20
Thin-film polarisers (×3, 22 CA 25, Comar Optics)	3 × £6
Hollow-shaft gimbal motor (Turnigy 4008)	£12
Electronic speed controller (DECS 50/5, Maxon)	£65 (cheaper options available)
32-bit microcontroller (Teensy 3.6, PJRC)	£29
Amplified photodiodes (x2, OPT101, Texas Instruments)	2 × £4.50
Light-to-frequency converter (TSL325R-LF, AMS)	£2
Total	**£300**

Table S1 Parts list for the polarimeter. The £300 cost is dominated by the expensive laser diode, which could be replaced by a low-cost laser pointer at < £15, bringing the build cost to £170. Further savings could be achieved by replacing the legacy ESC (a manual device with potentiometric control) by a modern ESC with PWM control (< £10). It would also be possible to amend the sDFT algorithm to use integer or fixed-point arithmetic, which would reduce the microcontroller cost to < £10 by removing the need for a Floating Point Unit. Making all three changes would bring the build cost below £100 without substantially reducing the performance of the polarimeter.

Manufacturer	Model	Quoted Accuracy	Quoted Precision	Quoted measurement times	Source of information
Perkin Elmer	Model 341	±0.002° for θ <1°<br>±0.2% for θ >1°	< 0.002°	0.1 – 100 s	https://www.perkinelmer.com/content/relatedmaterials/brochures/bro_series341343polarimeters.pdf
Anton Paar	MCP 150	±0.004°	±0.004°	< 12 s	https://www.anton-paar.com/uk-en/products/details/mcp-150-modular-compact-polarimeter/
Rudolph Research	AutoPol III	±0.002° for θ <1°<br>±0.2% for θ >1	±0.002°	< 5 s	https://rudolphresearch.com/products/polarimeters/autopol-iii/

Table S2 Quoted performance characteristics for typical high resolution polarimeters on sale in 2019.

Appendix S1 Brief derivation of the sliding DFT

Consider a digitised function $y(0), y(1), y(2), \dots y(N-1)$ obtained by sampling a continuous function $y(t)$ at the discrete times $t_0, t_1, t_2 \dots t_{N-1}$ where $t_i = i\Delta t$. The DFT decomposes the digitised signal into N complex oscillators of frequency $f_k = kf_s/N$, phase ϕ_k and real amplitude R_k, where $k = 0,1,2 \dots N-1$ and $f_s = 1/\Delta t$ is the sample rate. Defining the complex amplitude of each oscillator as $Y(K) = R_k e^{i\phi_k}$, the DFT may be written as a sequence of N complex numbers $Y(0), Y(1), Y(2), \dots Y(N-1)$ where:

$$Y(k) = R_k e^{i\phi_k} = \sum_{n=0}^{N-1} y(n) e^{-\frac{i2\pi kn}{N}} \tag{S1}$$

and $R_k = \mathrm{abs}(Y(k))$ and $\phi_k = \arg(Y(k))$. The sDFT is based on a sliding window of the N most recent data points. It follows from Eq. (S1) that, for a window starting at time $t = m$, the DFT is given by:

$$Y_m(k) = \sum_{n=0}^{N-1} y(m+n) e^{-\frac{i2\pi kn}{N}} \tag{S2}$$

Similarly, for a window starting at time $t = m+1$

$$Y_{m+1}(k) = \sum_{n=0}^{N-1} y(m+1+n) e^{-\frac{i2\pi kn}{N}} \tag{S3}$$

Substituting $n' = n+1$ into Eq. S3, we obtain:

$$\begin{aligned} Y_{m+1}(k) &= \sum_{n'=1}^{N} y(m+n') e^{-\frac{i2\pi k[n'-1]}{N}} \\ &= \sum_{n'=0}^{N-1} y(m+n') e^{-\frac{i2\pi k[n'-1]}{N}} - y(m) e^{\frac{i2\pi k}{N}} + y(m+N) e^{\frac{-i2\pi k[N-1]}{N}} \\ &= e^{\frac{i2\pi k}{N}} \left[\sum_{n'=0}^{N-1} y(m+n') e^{-\frac{i2\pi kn'}{N}} - y(m) + y(m+N) e^{-i2\pi k} \right] \end{aligned} \tag{S4}$$

Recognising the first term in brackets as $Y_m(k)$ and writing $e^{-i2\pi k} = 1$ (for integer values of k), we obtain the following recurrence formula for the k-th bin of the DFT:

$$Y_{m+1}(k) = e^{i2\pi k/N}[Y_m(k) - y(m) + y(m+N)] \tag{S5}$$

Hence, to advance the k-th bin of the DFT by one point in time, it is necessary to add the newest data point $y(m+N)$, subtract the oldest data point in the N-point window $y(m)$, and multiply the total by the phase factor $e^{i2\pi k/N}$.

Rounding errors in the calculation of the $e^{i2\pi k/N}$ pre-factor can lead to complex magnitudes slightly greater than one, causing the output to become unstable. Hence it is common to use a modified version of the sDFT: [1]

$$Y_{m+1}(k) = re^{i2\pi k/N}[Y_m(k) - r^N y(m) + y(m+N)] \tag{S6}$$

where r is a damping constant that is very slightly smaller than one. Other "absolutely stable" versions of the sDFT algorithm have been reported, [2] which do not require a damping constant and so avoid the (slight) associated numerical errors. However, they have a higher computational cost and were not required for the current application.

[1] R. G. Lyons, Understanding Digital Signal Processing (2nd Edition), Prentice Hall PTR, Upper Saddle River, NJ, USA, 2004.

[2] C. M. Orallo, I. Carugati, P. G. Donato, S. Maestri, Measurement 2015, 69, 9

Additional materials

Source code for the polarimeter, data files, and MATLAB code for Fig. 2 and Fig. S5, and design files for the PCBs and 3D-printed parts are available at:
https://doi.org/10.5281/zenodo.3571060

User comments

If you have any suggestions about how to improve the analytical performance of the polarimeter, enhance its functionality or reduce its build-cost, please get in contact via our wiki at https://github.com/ajharvie/polarimeter/wiki. Updates to the polarimeter will be published periodically.
